# Associations of *ADIPOQ* and *LEP* Gene Variants with Energy Intake: A Systematic Review

**DOI:** 10.3390/nu11040750

**Published:** 2019-03-30

**Authors:** Caroline Kroll, Silmara S.B.S. Mastroeni, Paul J. Veugelers, Marco F Mastroeni

**Affiliations:** 1 Post-graduate Program in Health and Environment, University of Joinville Region, Rua Paulo Malschitzki, 10, Joinville, Santa Catarina, CEP 89.219-710, Brazil; carolinekroll.bio@gmail.com; 2 Department of Health Sciences, University of Joinville Region, Rua Paulo Malschitzki, 10, Joinville, Santa Catarina, CEP 89.219-710, Brazil; silmara.mastroeni@univille.br; 3 Population Health Intervention Research Unit, School of Public Health, University of Alberta, 3-50 University Terrace, 8303–112 St, Edmonton, AB T6G 2T4, Canada; paul.veugelers@ualberta.ca

**Keywords:** *ADIPOQ*, *LEP*, energy intake, adipocytokines, leptin

## Abstract

This systematic review aims to evaluate the association of adiponectin (*ADIPOQ*) and leptin (*LEP*) gene variants with energy intake. Cross-sectional, cohort, and case–control studies that reported an association of leptin and/or adiponectin gene variants with energy intake were included in this review. Human studies without any age restrictions were considered eligible. Detailed individual search strategies were developed for each of the following bibliographic databases: Cochrane, Latin American and Caribbean Center on Health Sciences Information (LILACS), PubMed/MEDLINE, Scopus, and Web of Science. Risk of bias assessment was adapted from the Downs and Black scale and was used to evaluate the methodology of the included studies. Seven studies with a pooled population of 2343 subjects were included. The *LEP* and *ADIPOQ* gene variants studied were *LEP*-rs2167270 (*k* = 1), *LEP*-rs7799039 (*k* = 5), *ADIPOQ*-rs2241766 (*k* = 2), *ADIPOQ*-rs17300539 (*k* = 1), and *ADIPOQ* marker D3S1262 (*k* = 1). Two of the seven studies reviewed demonstrated a positive association between the *LEP*-rs7799039 polymorphism and energy intake. Two other studies—one involving a marker of the *ADIPOQ* gene and one examining the *ADIPOQ*-rs17300539 polymorphism—also reported associations with energy intake. More research is needed to further elucidate the contributions of genetic variants to energy metabolism.

## 1. Introduction

The prevalence of obesity has almost tripled over recent decades and represents a public health concern [1]. Obesity is the result of a chronic energy imbalance whereby energy intake exceeds energy expenditure [2,3]. There are different factors that lead to this energy imbalance and potentially to excess body weight, including access to low-cost foods that are low in nutritional value and high in energy, a sedentary lifestyle, eating behavior, and the increased consumption of processed and convenience foods and sugar-sweetened beverages, among others [4,5]. Some authors also indicate that total energy expenditure is driven by total daily activity, which is primarily determined by occupation [6]. However, in the last decade, some studies have demonstrated a genetic contribution to energy intake [7,8,9].

Among the different genes potentially associated with energy imbalance, genetic variants related to adipokines are of particular interest because of their effects on the endocrine system [10]. Adipokines comprise a large group of molecules, including hormones, cytokines, and growth factors that are produced and secreted by white adipose tissue [11]. Genetic variants related to adipokines and with hormonal effects are intrinsically associated with energy homeostasis and appetite regulation [7,12,13]. Among the different adipokines that may affect energy balance, specifically leptin and adiponectin play a key role in energy homeostasis and appetite regulation [14], particularly because they are the most abundant adipokines synthesized by adipose tissue [15]. Some studies have reported that genetic variants in adipokines genes, specifically the leptin (*LEP*) and adiponectin (*ADIPOQ*) genes, are associated with body mass index (BMI) and central adiposity [16,17,18,19,20].

It is well established that serum adiponectin concentrations are inversely proportional to the amount of fat tissue. Adiponectin acts at the hypothalamus and works to stimulate food intake [14]. The signaling pathway of adiponectin involves adenosine monophosphate kinase (AMPK) protein, which stimulates hunger [16,21,22]. Serum leptin concentrations, on the other hand, are positively associated with the amount of fat tissue. Leptin signals the hypothalamus that energy requirements are being met and that no more food intake is necessary [14]. Leptin also activates anorexigenic and orexigenic neurons that are responsible for the inhibition and stimulation of feeding, respectively [17,18,19]. Increasing leptin and decreasing adiponectin concentrations may reduce appetite and caloric intake and thus result in a more negative energy balance [14].

Although several studies have demonstrated an association of the *ADIPOQ* and *LEP* genes with BMI [16,23,24,25,26,27,28,29,30,31], there is no consensus regarding the influence of these genes on energy intake and no systematic reviews or meta-analyses exist on the topic. Therefore, this systematic review aims to evaluate the available evidence for associations of *ADIPOQ* and *LEP* gene variants with energy intake.

## 2. Methods

This systematic review was conducted using the Preferred Reporting Items for Systematic Reviews and Meta-Analyses (PRISMA) checklist [32].

### 2.1. Protocol and Registration

The protocol of this systematic review was registered at the Centre for Reviews and Dissemination (CRD), international prospective register of systematic reviews (PROSPERO).

### 2.2. Eligibility Criteria

Cross-sectional, cohort, and case–control studies that reported an association of leptin and/or adiponectin gene variants with energy intake (kcal or kJ) were included in this review. Human studies without any age restrictions were considered eligible. We excluded studies that reported other genes or other molecular conditions or outcomes other than energy intake (kcal or kJ). Reviews, letters, personal opinions, book chapters, or conference abstracts were not considered in this systematic review.

### 2.3. Information Sources and Search Strategy

Detailed individual search strategies were developed for each of the following bibliographic databases: Cochrane, LILACS, PubMed/MEDLINE, Scopus, and Web of Science (Appendix A). A grey literature search was undertaken using Google Scholar. The grey literature refers to unpublished research or published in non-commercial form and allows a more comprehensive search strategy [33]. The reference list of the studies included was searched manually to identify additional references. We conducted all searches from beginning dates and without year/period restriction until 8 October 2018. Duplicate hits were removed and references were managed using EndNote (EndNote; Thomson Reuters, Philadelphia, PA, USA).

### 2.4. Study Selection

The articles were selected in two steps. In the first step, two authors (C.K. and S.S.B.S.M.) independently read the titles and abstracts of all identified articles. These authors selected the articles that met the inclusion criteria based on the article’s title and abstract. Studies that did not meet these inclusion criteria were discarded. In the second step, the same two authors read the full articles and excluded studies that did not meet the inclusion criteria. Finally, two other authors (M.F.M. and P.J.V.) participated in the selection when disagreements emerged between the first two authors (C.K. and S.S.B.S.M.).

### 2.5. Data Collection Process and Data Items

Data collection was performed in the same way as described in the previous section. Two authors (C.K. and S.S.B.S.M.) independently collected the required information (authors, year of publication, study design, sample size, sample characteristics, *LEP*/*ADIPOQ* gene variants, energy intake assessment, and main conclusion) from the selected articles. Disagreements were resolved by discussion between the two review authors; if no agreement could be reached, a third author (M.F.M.) was consulted.

### 2.6. Risk of Bias in Individual Studies

Two authors (S.S.B.S.M. and C.K.) were responsible for reviewing the methodological quality and for assessing the risk of bias according to the scale adapted from Downs and Black [34]. According to these criteria [34], the articles were grouped into three categories: (a) first category: articles with a cohort methodological design, with a maximum score of 19; (b) second category: randomized crossover trials with a maximum score of 20, and (c) third category: case-control studies with a maximum score of 28. To ensure the proportion of results between studies, the score obtained for each article was divided by the maximum possible score for each one and multiplied by 100. The median scores were calculated. Articles with a score below the median were classified as having a high risk of bias.

### 2.7. Summary Measures

All outcome measurements related to energy intake were considered in this review: mean intake (kcal or kJ/day), relative risk, and odds ratios. The outcomes were evaluated according to the risk alleles or genotypes.

## 3. Results

### 3.1. Study Selection

**Figure 1:**
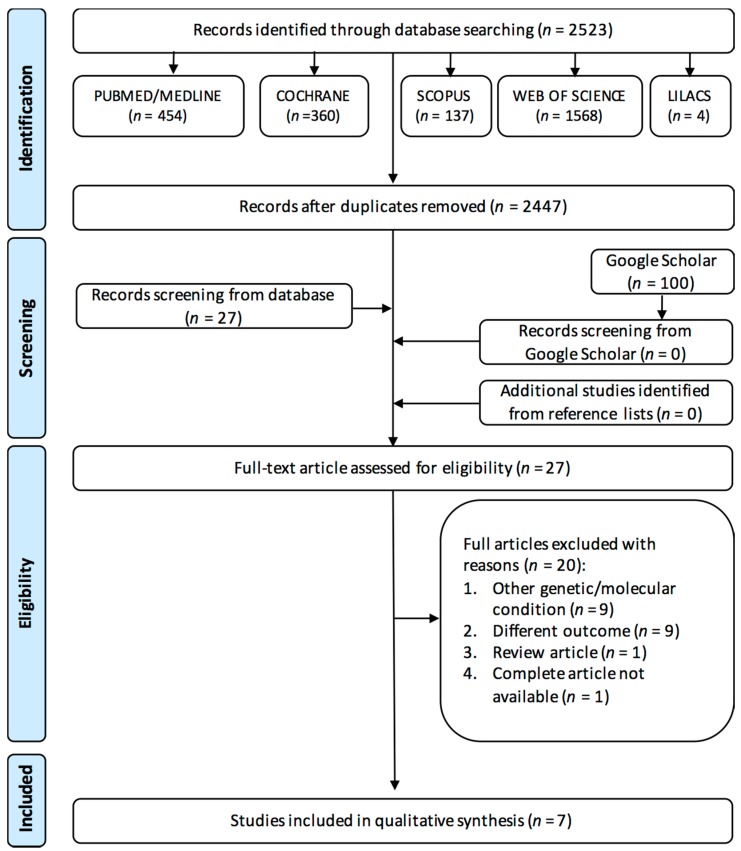
Flow diagram of study selection adapted from Preferred Reporting Items for Systematic Reviews and Meta-Analyses (PRISMA) 2. LILACS: Latin American and Caribbean Center on Health Sciences Information.

We conducted this systematic review in two steps: in step 1, the electronic search retrieved 2523 citations. After the removal of duplicates, 2447 articles were identified. Comprehensive evaluation of titles and abstracts led to the exclusion of 2420 articles, resulting in 27 articles in the first step. No additional articles were identified after checking the reference lists. Google Scholar searches of the first 100 results were performed, but no additional articles were identified. As part of step 2, we read the full text of the 27 articles and excluded 20 (Appendix B), leaving seven studies for the systematic review. A flow chart detailing the process of identification, inclusion, and exclusion of studies is depicted in Figure 1.

### 3.2. Study Characteristics

The seven studies originated from five countries: the Czech Republic, Tunisia, Canada, the United Kingdom, and Brazil. All studies were written in English and published after 2008. Three studies were cohort studies, three were case-control studies, and one was a randomized crossover trial. Most studies involved adults, including only men in one and only pregnant women in another. One study was conducted on children. The pooled population included in this systematic review was 2343 subjects. The *LEP* and *ADIPOQ* gene variants studied were *LEP*-rs2167270 (*k* = 1), *LEP*-rs7799039 (*k* = 5), *ADIPOQ*-rs2241766 (*k* = 2), *ADIPOQ*-rs17300539 (*k* = 1), and *ADIPOQ* marker D3S1262 (*k* = 1). Most of the studies used a food frequency questionnaire (*k* = 5) to assess dietary intake, one used dietary recall, and one used an ad libitum lunch offered 90 min after dairy snacks. The descriptive characteristics of the seven studies are summarized in Table 1.

### 3.3. Risk of Bias within Studies

The risk of bias according to the criteria of Downs and Black [34] is shown in Table 1. A median score of 73.9% was obtained, with a maximum score of 100.0% and a minimum score of 68.4%. Four articles were considered to have a low risk of bias [23,38,39,40]. Three studies showed values below the median score and were therefore considered to have a high risk of bias and reduced methodological quality [35,36,37].

### 3.4. Synthesis of Results

Since the data of the included studies showed notable and significant heterogeneity, a meta-analysis was not justified. Therefore, only a qualitative synthesis could be performed.

#### 3.4.1. LEP-rs7799039

A case-control study conducted in Tunisia demonstrated that adults carrying the AA genotype of the *LEP*-rs7799039 polymorphism had significantly higher daily energy intake (*p* = 0.048; GG: 2853 ± 1215 kcal; GA: 2889 ± 1277 kcal; AA: 3431 ± 1609 kcal) [23]. In a cohort study [39] among pregnant women in Brazil, the authors showed a significant association between allele A of *LEP*-rs7799039 and the change in energy intake from pre-pregnancy to pregnancy, with carriers of the allele having lower total mean adjusted energy intake (GA+AA = 1964 kcal/day, 95% CI: 1684–2290 kcal/day; GG = 2192 kcal/day, 95% CI: 1890–2542 kcal/day; *p* = 0.04). However, a randomized crossover trial among English men detected no effect of the *LEP*-rs7799039 polymorphism on ad libitum energy intake at lunch (AA = 3744 ± 391 kJ; GG+GA = 4222 ± 285 kJ; *p* = 0.290) [38]. Furthermore, no associations between the *LEP*-rs7799039 polymorphism and total energy intake were found in a prospective cohort of Brazilian children [40] and in a case-control study of a Czech population [36].

#### 3.4.2. LEP-rs2167270

One case-control study [35] conducted on Czech adults found that the *LEP*-rs2167270 polymorphism did not serve as an independent predictor of daily energy intake. However, considering the distribution of energy intake during the day, *LEP*-rs2167270 was associated with the energy value of supper (β = 0.13; *p* = 0.05), with subjects carrying the AG genotype expressing a tendency toward high energy intake from supper.

#### 3.4.3. ADIPOQ-rs2241766

In the same Czech case-control study [35], the *ADIPOQ*-rs2241766 polymorphism was not a predictor of daily energy intake. However, *ADIPOQ*-rs2241766 was associated with energy from breakfast (β = 0.15; *p* = 0.02). Another case-control study conducted by the same research group from the Czech Republic [36] reported no associations of *ADIPOQ*-rs2241766 with total energy intake.

#### 3.4.4. ADIPOQ-rs17300539

A prospective cohort study [40] among Brazilian children aged 12 to 16 months found no significant associations of the *ADIPOQ*-rs17300539 polymorphism with energy intake. However, at one year of age, children carrying allele A had lower total energy intake/day than G/G homozygotes (G = 952 ± 387 kcal; A = 841 ± 386 kcal; *p* = 0.045). At four years of age the authors observed the opposite: G-carriers had higher total energy intake/day than A-carriers, but these differences were not statistically significant (G = 1501 kcal; A = 1588 kcal; *p* = 0.149). Moreover, the authors reported strong linkage disequilibrium between *ADIPOQ*-rs17300539 and *ADIPOQ*-rs266729, suggesting that the two polymorphisms exert the same effect on energy intake, regardless of whether they are analyzed alone or together.

#### 3.4.5. ADIPOQ gene marker D3S1262

A cohort study among French-Canadians that examined a marker (D3S1262) of chromosome 3q27.3 in a region harboring the adiponectin gene demonstrated significant linkage between this marker and energy intake (logarithm of odds (LOD) score: 2.24, *p* < 0.01) [37]. The authors defined a LOD threshold ≥ 1.75 as suggestive of linkage (*p* < 0.01).

## 4. Discussion

### 4.1. Summary of Evidence

To our knowledge, this is the first systematic review addressing associations of *ADIPOQ* and *LEP* gene variants with energy intake. Two of the seven studies reviewed demonstrated a positive association of the *LEP*-rs7799039 polymorphism [23,39] with energy intake. One study reported that Tunisians carrying the AA genotype had a higher energy intake [23], whereas another study suggested that pregnant Brazilian women with the A-risk allele have a lower energy intake than G-carriers [39]. Although the *LEP*-rs7799039 polymorphism has been associated with energy intake in two studies, four other studies found no association of these polymorphisms with energy intake [35,36,38,40]. Two other studies—one involving a marker of the *ADIPOQ* gene and one examining the *ADIPOQ*-rs17300539 polymorphism—also reported associations with energy intake [37,40]. Another study reported a significant association of allele G of the *ADIPOQ*-rs17300539 polymorphism with higher energy intake in one-year-old children when compared with those carrying the A-risk allele [40]. No association with energy intake was observed for the *ADIPOQ*-rs2241766 or *LEP*-rs2167270 polymorphism.

The *LEP*-rs7799039 polymorphism (*LEP*-2548 G/A) consists of a guanine to adenine substitution in the gene promoter region [23]. This mutation affects gene expression, probably at the transcriptional level, altering the levels of leptin secreted by adipose tissue and consequently disturbing satiety control [24,25]. Leptin is a satiety hormone that acts on anorexigenic (appetite-decreasing) neurons and provides signals to the hypothalamus, decreasing appetite and increasing energy expenditure [18,19,41,42]. However, the findings of studies regarding the risk allele are controversial. Some authors suggest that the G-allele is the risk allele for developing excess body weight [26,27,43], while others indicate the A-allele to be associated with excess body weight [23,28,44]. These controversial results are not well elucidated in the literature. The different findings may be due to the diversity in the genetic background of the world population, but may also be attributed to interactions with other parts of leptin and/or leptin receptor genes [45]. We recommend that further studies should be conducted that seek to explain this divergence concerning the risk allele.

The *ADIPOQ* gene is associated with the expression of adiponectin, an adipocytokine abundantly produced by adipose tissue [20,46]. Adiponectin participates in energy homeostasis and acts on adipose tissue [22,47]. Decreases in adiponectin concentrations promote the phosphorylation of target proteins by AMPK, which is critical for lipid and carbohydrate metabolism. As a consequence, the metabolism of many tissues is affected, resulting in distancing of energy-consuming processes and stimulating food consumption to provide energy. Some mutations in the *ADIPOQ* gene can alter the expression of the gene, and thus affect energy homeostasis and weight status. One of the genetic variants related to this process is the rs17300539 polymorphism (*APM1*-11391G>A) located in the promoter region of the gene, which consists of a guanine to adenine substitution. The A-allele of *ADIPOQ*-rs17300539 seems to increase adiponectin levels due to higher transcription of the *ADIPOQ* gene [48], suggesting an influence of this variant on obesity-related traits in both adults and children [40,49].

Although the genetic variants in the *ADIPOQ* and *LEP* genes have important effects on energy intake and weight gain, we acknowledge the role of the environment and its influence on obesogenic processes. The genetic inheritance pattern most commonly associated with obesity is polygenic inheritance (95%) [50], which is the result of multiple genetic interactions that lead to the manifestation of obesity. This pattern of inheritance establishes that each gene associated with obesity has a small contribution to this phenotype [2,51]. However, a set of loci play an important role in determining the individual’s weight status and how it is influenced by environmental factors such as diet and physical activity [2,51]. Therefore, understanding the genetic and environmental mechanisms involved in appetite regulation, energy metabolism, and fat storage may contribute to the development of potential interventions to prevent future health problems mainly related to weight status.

This systematic review indicates that we need more studies with a large number of individuals to examine the association between energy intake and genetic variants, especially variants of the *LEP* and *ADIPOQ* genes. As these genes are strongly associated with weight status at any age, the genetic and dietary patterns should be analyzed concurrently to better understand the rise in excess body weight. Determining which aspect(s) of the energy balance equation (carbohydrate, lipid, or protein) is influenced by the genetic variant is another important research area that needs more attention.

### 4.2. Limitations

Several methodological limitations of this review should be considered. First, only seven articles met the inclusion criteria. Our inclusion and exclusion criteria may have excluded a considerable number of studies; however, we expect this number to be small because relatively few studies currently exist in this area. Second, our search strategy may not have retrieved the total body of literature despite consultation with a professional librarian and the use of a comprehensive set of search terms. Third, the relatively few studies retrieved from the literature were very heterogeneous and modest in size, making it challenging to compare them. Fourth, the studies used different instruments for quantifying energy intake. Most studies applied self-report methods (food frequency questionnaires) for quantifying dietary and energy intake. Since this method is subject to recall bias, among other biases, future research should develop more accurate methods for assessing energy intake. Finally, some general limitations of genetic association studies apply to this review: minor frequency alleles in studies with small sample sizes often lead a lack of statistical power to demonstrate a conclusive result.

## Figures and Tables

**Table 1:** Summary of descriptive characteristics of the included studies.

Author, Year	Country, Ethnicity	Study Design	Sample Size, *n*	Sample Characteristics	*LEP*/*ADIPOQ* Gene Variants	Energy Intake	Main Conclusion	Risk of Bias *
Bienertova-Vasku, Bienert et al., 2010 [35]	Czech Republic, Caucasian	Case-control study	409	Obese (*n* = 252) and normal-weight (*n* = 157) groups	*LEP*-rs2167270 (*LEP* + 19A/G); *ADIPOQ*-rs2241766 (*ADIPOQ* 45T/G, *ADIPOQ* 94T/G)	Standardized 7-day food records	None of the examined polymorphisms served as an independent predictor for the percentage of daily energy intake. The *ADIPOQ*-rs2241766 polymorphism was associated with the energy value of breakfast, defined as the first meal during the day (β = 0.15; *p* = 0.02). Moreover, the *LEP*-rs2167270 polymorphism was correlated with the energy value of supper (β = 0.13; *p* = 0.05), with AG heterozygotes expressing a tendency toward the highest energy intake in their supper.	72.7
Bienertova-Vasku, Bienert et al., 2008 [36]	Czech Republic, Caucasian	Case-control study	185	Obese (*n* = 125) and normal-weight (*n* = 60) groups	*LEP*-rs7799039 (*LEP*-2548 G/A); *ADIPOQ*-rs2241766 (*APM1* T94G)	7-day food records, total energy	None of the examined polymorphisms were associated with total energy intake.	72.7
Boumaiza, Omezzine et al., 2012 [23]	Tunisia	Case-control	329	Obese (*n* = 160) and non-obese (*n* = 169) groups	*LEP*-rs7799039 (*LEP* G2548A)	3-day dietary record: 2 weekdays and 1 weekend day	The AA genotype had significantly higher daily energy intake. GG: 2853 ± 1215 kcal; GA: 2889 ± 1277 kcal; AA: 3431 ± 1609 kcal; *p* = 0.048	77.3
Choquette, Lemieux et al., 2008 [37]	French-Canadian subjects	Cohort	836	Random sampling and ascertainment through obese probands	Marker D3S1262 on chromosome 3q27.3—region harboring the *ADIPOQ* gene	3-day dietary record: 2 weekdays and 1 weekend day	A significant linkage was observed on chromosome 3q27.3 with marker D3S1262. LOD score: 2.24, *p* < 0.0001	68.4
Dougkas, Yaqoob et al., 2013 [38]	United Kingdom	Randomized crossover trial	40	Healthy, non-smoking, overweight men aged 18–50 years with a BMI of 25.0–29.9 kg/m^2^	*LEP*-rs7799039	Energy intake was assessed by an ad libitum lunch offered 90 min after dairy snacks or water control	rs7799039 did not have an effect on ad libitum energy intake at lunch. AA: 3744 ± 391 kJ; GG+GA: 4222 ± 285; *p* = 0.290	75.0
Martins, Trujillo et al., 2018 [39]	Brazil	Cohort	220	Pregnant	*LEP*-rs7799039	Semi-quantitative FFQ: consumption frequency of food items over the 6 months before the interview. Data collected at 5–13 weeks of gestation covered the pre-pregnancy period, and data collected at 30–36 weeks of gestation refers to dietary intake of women during pregnancy	There was a significant association between allele A of *LEP*-rs7799039 and change in dietary intake from pre-pregnancy to pregnancy, with individuals presenting lower total energy intake. GA+AA: 1964 kcal/day, 95%CI: 1684–2290 kcal/day; GG: 2192 kcal/day, 95%CI: 1890–2542 kcal/day; *p* = 0.04	100.0
Zandona, Rodrigues et al., 2013 [40]	Brazil	Prospective cohort study	325	Children at 12–16 months and at 3–4 years of age	*ADIPOQ*-rs17300539 (*APM1* -11391G>A); *ADIPOQ*- rs266729 (*APM1*-11377C>G); *LEP*-rs7799039 (*LEP*-2548G>A)	12–16 months: 24-h diet recall to record the child’s food intake on the day before the home visit 3–4 years: two 24-h diet recalls were collected on two randomly selected and non-consecutive days	There was a strong linkage disequilibrium between *ADIPOQ*-rs17300539 and *ADIPOQ*-rs266729. Children carrying the *ADIPOQ*-rs17300539 A-allele had lower total energy intake/day than G/G homozygotes at 1 year. G: 952 ± 387 kcal; A: 841 ± 386 kcal; *p* = 0.045. The opposite effect was observed at 4 years of age: G-carriers had higher total energy intake/day than A-carriers, but without statistical significance. G: 1501 kcal; A: 1588 kcal; *p* = 0.149. Moreover, no associations were observed between *LEP*-rs7799039 and dietary parameters.	95.5

LOD: logarithm of the odds; BMI: body mass index; FFQ: food frequency questionnaire. * Median risk of bias = 73.9.

## Appendix A. Search Strategy

**Table:**

Database	Search
PubMed/MEDLINE (8 October 2018)	Search ((((((((((ADIPOQ gene)[Title/Abstract] OR adiponectin gene)[Title/Abstract] OR ACDC gene)[Title/Abstract] OR ADPN gene)[Title/Abstract] OR AMP1 gene)[Title/Abstract] OR APM-1 gene)[Title/Abstract] OR GBP28 gene)[Title/Abstract] OR ACRP30 gene [Title/Abstract])) OR (((((LEP gene)[Title/Abstract] OR leptin gene)[Title/Abstract] OR OB gene)[Title/Abstract] OR OBS gene)[Title/Abstract] OR LEPD gene [Title/Abstract])) AND energy intake Title/Abstract] Sort by: Best Match Filters: Humans
Scopus ^a,b^ (8 October 2018)	(TITLE-ABS-KEY (lep AND gene OR leptin AND gene OR ob AND gene OR obs AND gene OR lepd AND gene) OR TITLE-ABS-KEY (adipoq AND gene OR adiponectin AND gene OR acdc AND gene OR adpn AND gene OR apm1 AND gene OR apm-1 AND gene OR gbp28 AND gene OR acrp30 AND gene) AND TITLE-ABS-KEY (energy AND intake))
Web of Science ^b^ (8 October 2018)	TITLE: (LEP gene OR leptin gene OR OB gene OR OBS gene OR LEPD gene) OR TITLE: (ADIPOQ gene OR adiponectin gene OR ACDC gene OR ADPN gene OR APM1 gene OR APM-1 gene OR GBP28 gene OR ACRP30 gene) AND TITLE: (energy intake)
Cochrane ^a^ (8 October 2018)	LEP gene OR leptin gene OR OB gene OR OBS gene OR LEPD gene in Title Abstract Keyword OR ADIPOQ gene OR adiponectin gene OR ACDC gene OR ADPN gene OR APM1 gene OR APM-1 gene OR GBP28 gene OR ACRP30 gene in Title Abstract Keyword AND energy intake in Title Abstract Keyword - (Word variations were searched)
LILACS (8 October 2018)	(tw: (LEP gene OR leptin gene OR OB gene OR OBS gene OR LEPD gene)) OR (tw: (ADIPOQ gene OR adiponectin gene OR ACDC gene OR ADPN gene OR APM1 gene OR APM-1 gene OR GBP28 gene OR ACRP30 gene)) AND (tw: (energy intake OR consumo energético OR ingestão calórica))
Google Scholar (17 January 2017)	ADIPOQ gene OR LEP gene and energy intake (without patents or citations)

^a^ Refined by “title, abstract, and keywords”. ^b^ Refined by “Article” and/or “Article in press”.

## Appendix B. List of Excluded Articles

**Table:**

Article	Reason
Mariman et al. (2015) [52]	1
Brunkwall et al. (2016) [53]	1
Alharbi et al. (2014) [54]	1
Alharbi et al. (2014) [54], Liu et al. (2003) [55]	1
Li et al. (2014) [56]	2
Feitosa et al. (2000) [57]	1
Collaku et al. (2004) [58]	1
Dubois et al. (2013) [59]	1
Celis-Morales et al. (2017) [60]	1
Mizuta et al. (2008) [61]	2
de Krom et al. (2007) [62]	2
Trayhum et al. (1996) [63]	3
Valladares et al. (2014) [64]	2
de Luis et al. (2018) [65]	2
Gajewska et al. (2016) [66]	2
Licinio et al. (2007) [67]	1
Mammes et al. (1998) [68]	2
Park et al. (2014) [69]	2
Faith et al. (1999) [70]	4

1: Other genetic/molecular condition, *n* = 9; 2: Different outcome, *n* = 9; 3: Review article, *n* = 1; 4: Complete article not available, *n* = 1.
